# Entropy Generation Optimization for Rarified Nanofluid Flows in a Square Cavity with Two Fins at the Hot Wall

**DOI:** 10.3390/e21020103

**Published:** 2019-01-22

**Authors:** Wael Al-Kouz, Ahmad Al-Muhtady, Wahib Owhaib, Sameer Al-Dahidi, Montasir Hader, Rama Abu-Alghanam

**Affiliations:** 1 Mechatronics Engineering Department, German Jordanian University, Amman 11180, Jordan; 2 Mechanical and Maintenance Engineering Department, German Jordanian University, Amman 11180, Jordan; 3 Aeronautical Engineering Department, Jordan University of Science and Technology, Irbid 22110, Jordan; 4 Energy Engineering Department, German Jordanian University, Amman 11180, Jordan

**Keywords:** natural convection, entropy generation, square cavity, low pressure, nanofluid

## Abstract

Computational Fluid Dynamics (CFD) is utilized to study entropy generation for the rarefied steady state laminar 2-D flow of air-Al_2_O_3_ nanofluid in a square cavity equipped with two solid fins at the hot wall. Such flows are of great importance in industrial applications, such as the cooling of electronic equipment and nuclear reactors. In this current study, effects of the Knudsen number (*Kn*), Rayleigh number (*Ra*) and the nano solid particle’s volume fraction (ϕ) on entropy generation were investigated. The values of the parameters considered in this work were as follows: 0≤Kn≤0.1, $10^{3}\leq Ra\leq 10^{6},\;0\leq \phi \leq 0.2$. The length of the fins (L_F_) was considered to be fixed and equal to 0.5 m, whereas the location of the fins with respect to the lower wall (*H_F_*) was set to 0.25 and 0.75 m. Simulations demonstrated that there was an inverse direct effect of *Kn* on the entropy generation. Moreover, it was found that when *Ra* was less than 10^4^, the entropy generation, due to the flow, increased as ϕ increases. In addition, the entropy generation due to the flow will decrease at *Ra* greater than 10^4^ as ϕ increases. Moreover, the entropy generation due to heat will increase as both the ϕ and *Ra* increase. In addition, a correlation model of the total entropy generation as a function of all of the investigated parameters in this study was proposed. Finally, an optimization technique was adapted to find out the conditions at which the total entropy generation was minimized.

## 1. Introduction

Unconventional reservoirs have drawn intensive attention recently [1], and fractal-based approaches are key methods used to characterize the pore structure, physical properties, and fluid flow in them under different mechanisms [2,3,4]. One of the basic problems that has been investigated deeply in the last few decades is the natural convection mode of heat transfer that serves in a number of engineering applications, for example solar collectors [5,6], fuel cell industry [7], petroleum engineering [8,9], and cooling of electronic components [10,11], etc. The unsatisfying heat-transfer rate due to the natural convection, however, is a significant issue for the application. As a result, the dispersion of nano solid particles into a base fluid has been developed as a widely-used method to address such an issue. By dispersing nano solid particles into the base fluid, the resulting nanofluid will have superior thermal properties compared to the base fluid. For instance, Choi et al. [12] introduced “nanofluids” used in many industrial applications. In the work conducted by Khanafer et al. [13], the numerical solution of natural convection heat transfer in a two-dimensional enclosure where nanofluid is used as the working fluid was analyzed. They concluded that there is a direct proportional relationship between the heat transfer rate and the ϕ at a given Grashof number. Moreover, Khanafer et al. [14] studied the validity of nanofluid’s effective viscosity and thermal conductivity models along with experimental results available in the literature and their features in the enhancement of heat transfer. Buongiorno [15] discussed and provided an explanation for the convective heat transfer enhancement associated with using nanofluid. He proposed a new model for the transport phenomena in nanofluids based on a two-component nonhomogeneous equilibrium model. In Oztop et al. [16], Computational Fluid Dynamics (CFD) analysis was used to solve a mathematical model for fluid flow and heat transfer due to buoyancy effect in a partially heated cavity filled with nanofluid. They noticed that at a given *Ra*, there was an enhancement in heat transfer as *ϕ* increased. In addition, Ghasemi et al. [17] numerically studied the natural convection of water/Al_2_O_3_ nanofluid in a square cavity under a magnetic field. They found that for any *ϕ*, heat transfer rate was strongly dependent on *Ra*; it may enhance or deteriorate. Also, Kefayati et al. [18] simulated the heat transfer and flow of free convection in cavities filled with water/SiO_2_ using a lattice Boltzmann method. They concluded that there was a direct relationship between the heat transfer rate and *ϕ* for the studied aspect ratios and *Ra*. Additionally, Kefayati [19] analyzed entropy generation and heat transfer of laminar free convection flow in a porous square cavity filled with non-Newtonian nanofluid Cu/water using a finite difference lattice Boltzmann method. He found that the heat transfer rate was enhanced and entropy generation was dropped when both *ϕ* and *Ra* were increased. Al-Kouz et al. [20] numerically investigated free convection heat transfer characteristics of rarefied flows in an inclined square enclosure equipped with two solid or porous fins at the hot wall. They found that with equipped fins at the hot wall, the heat transfer rate was enhanced. Moreover, they found that using porous fins had an advanced impact on heat transfer. Al-Kouz et al. [21] numerically studied the low-pressure gaseous flows’ free convection heat transfer characteristics of nanofluid (Air/Al_2_O_3_) inside a square enclosure equipped with two solid fins at the hot wall. They revealed that for a given *Ra*, adding nanoparticles resulted in an enhancement in the heat transfer rate.

Studying the rate of entropy generation is important in engineering because it suitably calculates the irreversibility of thermodynamics. For example, Kefayati et al. [22] analyzed the natural convection flow in an inclined cavity of non-Newtonian nanofluid using Buongiorno’s mathematical model by the finite difference lattice Boltzmann method. They observed that the lowest entropy generation and highest Bejan number occur at inclined an angle of zero at a given *Ra*. Parvin et al. [23] numerically investigated entropy generation and laminar free convective heat transfer in an odd-shaped enclosure filled with Cu/water nanofluid. Their results revealed that with increasing *Ra*, entropy generation caused by heat was increased while the entropy generation caused by the fluid flow was decreased. They also extracted the optimum value of *Ra* at which the heat transfer was maximized and the total entropy generation was minimized. In the work of Merji et al. [24] and Mahmoudi et al. [25], numerical study using the lattice Boltzmann method for the laminar free convection and entropy generation in a square enclosure filled with water/Al_2_O_3_ nanofluid under a magnetic field was conducted. They found that *ϕ* had a direct effect on the heat transfer rate and an inverse effect on the total entropy generation. In the article by Armaghani et al. [26], a numerical study of the entropy generation and natural convection heat transfer in a baffled L-shaped cavity filled with water-Alumina nanofluid was presented. The authors revealed that as the aspect ratio increased the heat transfer rate enhanced, particularly when nanofluid was utilized as a working fluid. Al-Zamily [27] numerically studied the influence of a porous central layer thickness inside a cavity on heat transfer, fluid flow and entropy generation. The cavity was filled with nanofluid (water/ TiO_2_) at a constant wall heat flux located at two different wall positions. Results showed that the nanofluid flow was stronger and heat transfer rate increased as the central porous layer thickness decreased. He also concluded that the heat transfer rate was enhanced with *ϕ*. Bouchouch et al. [28] investigated the free convection heat transfer and entropy generation of nanofluid (water/Al_2_O_3_) in a square enclosure with a thick bottom wall heated with a non-isothermal heater with a sinusoidal function. The authors showed that using the nanofluid enhanced the heat transfer. Moreover, they concluded that the entropy generation increased with *Ra*. Ashorynejad et al. [29] numerically investigated the entropy generation and free convection heat transfer in a square porous enclosure with various porosities filled with different water base nanofluids (Al_2_O_3_, TiO_2_ and CuO) using the lattice Boltzmann method. They concluded that the dispersion of nano solid particles decreased the total entropy generation and enhanced heat transfer. They also concluded that the entropy generation was increased with cavity porosity. In their work, Sheremet et al. [30] numerically studied the free convection heat transfer and entropy generation of water based nanofluid inside a square cavity with variable temperature distribution sidewalls. They concluded that the total entropy generation increased with *Ra* and a rise of the temperature distribution in the sidewalls. Alsabery et al. [31] numerically investigated the free convection and entropy generation of nanofluid (water/Al_2_O_3_) in a square enclosure with concentric solid inserts at different temperature distributions. They observed a strong heat transfer rate enhancement with increasing *Ra* for a given Rayleigh number range. In addition, they concluded that the total entropy generation rose with increasing *Ra* and with the reduction in the size of the concentric solid insert beyond a given *Ra*. A numerical investigation using the two-phase mixture and Darcy-Birnkman-Forchheimer model for free convection and entropy generation of nanofluid (water/Cu) inside a cavity furnished with porous fins was presented by Siavashi et al. [32]. They revealed that a low ϕ enhanced the heat transfer rate at a given *Ra*. They also found that the thermal irreversibility was dominant pertaining to entropy generation due to friction. Finally, they concluded that the entropy generation was reduced by using porous fins. Kashyap et al. [33] numerically investigated using a two-phase lattice Boltzmann the natural convection of nanofluid (water/Cu) in a porous square cavity at different boundary conditions. They observed that for all the boundary conditions they studied, the use of nanofluid enhancesd the heat transfer and reduced the entropy generation depending on ϕ. Gibanov et al. [34] analyzed numerically the free convection heat transfer and entropy generation of nanofluid (water/Alumina) in a lid-driven cavity with a bottom solid wall. They concluded that ϕ had a direct effect on the heat transfer. Mansour et al. [35] numerically investigated the entropy generation and magneto-hydrodynamics (MHD) natural convection heat transfer in a square porous enclosure filled with hybrid nanofluid (water/Cu/Al_2_O_3_). They revealed that for a given *Ra*, the heat transfer rate was decreased and the entropy generation was increased with increasing *ϕ*. Rahimi et al. [36] investigated natural convection heat transfer and the entropy generation of nanofluid (water/CuO) inside a square cavity equipped with fins. They concluded that the heat transfer rate increased with increasing *Ra* and *ϕ*, whereas entropy generation increased with *Ra* and decreased with *ϕ* for the investigated parameters ranges. In their paper, Rashidi et al. [37] investigated the effects of different modeling approaches on the entropy generation in a circular tube heat exchanger using nanofluids, where the considered geometry was a horizontal tube with a constant wall heat flux. The flow regime was turbulent. They found out that the values for entropy generation were very close for the single phase and mixture models. Additionally, they concluded that for the higher volume fractions (i.e., greater than 4%), differences between the models appeared. In their work, Yarmand et al. [38], numerically studied the entropy generation during turbulent flow of Zirconia/water and other nanofluids in a square cross section tube with a constant heat flux, where the flow was assumed to be turbulent. Their results showed that with the optimal volume concentration of nanoparticles minimized, the entropy generation increased when Reynolds number decreased. It was also found that the thermal entropy generation increased with the increase of the nanoparticle size, whereas the frictional entropy generation decreased. Entropy generation in the thermal radiative loading of structures with distinct heaters has been studied numerically by Jamalabadi et al. [39]. They used a finite volume analysis and the semi implicit method for pressure linked equations to solve for the continuity, momentum and energy equations, and their results showed that the entropy value was more influenced by the temperature than the density. They also showed that the heating ratio of the onset of natural and radiative entropy generation increased by an increase of number of discrete heater sources. In their research, Aghaei et al. [40] experimentally and numerically analyzed the effect of horizontal and vertical elliptic baffles inside an enclosure on the mixed convection of a MWCNTs-water nanofluid and its entropy generation, and they concluded that the horizontal placement of a thermal baffle led to a higher heat transfer rate. Moreover, they found that the entropy generation values in the horizontal position were higher than the vertical position. Mahmoudinezhad et al. [41] numerically and experimentally investigated the adiabatic partition effect on the natural convection heat transfer inside a square cavity, where the flow was considered to be steady state, 2-D. They used a finite volume analysis along with the Mach–Zehender interferometer to carry out the study. Their results showed that the average Nusselt number increased with an increasing Rayleigh number. However, for a given *Ra*, the maximum and minimum heat transfer occurred at the partition angles of 45° and 90°, respectively. Finally, Nasiri et al. [42] used a smoothed particle hydrodynamics approach to investigate the forced convection nanofluid heat transfer over a horizontal cylinder. Their results show that the smoothed particle hydrodynamics approach was the appropriate method for such numerical modeling. In addition, they concluded that the nanofluid heat transfer characteristics had marked improvements compared to base fluids.

Despite many studies in the field of entropy generation dealing with water-based nanofluid inside square cavities, there is a lack in studies that tackle the entropy generation of natural convection low-pressure cavities filled with an air based nanofluid. Therefore, the purpose of the present numerical investigation is to give more insight into the entropy generation in square cavities equipped with two solid fins at the hot wall filled with low-pressure air/Al_2_O_3_ nanofluid. Analyzed parameters include the Rayleigh number (10^3^ ≤ *Ra* ≤ 10^6^) to cover both the conduction dominant and convection dominant modes of heat transfer, the Knudsen number (0 ≤ *Kn* ≤ 0.1) to cover both the slip and continuum flow regimes, and the nanosolid particles volume fraction (0 ≤ *ϕ* ≤ 0.2).

## 2. Mathematical Modeling

### 2.1. Mathematical Formulation

**Figure 1:**
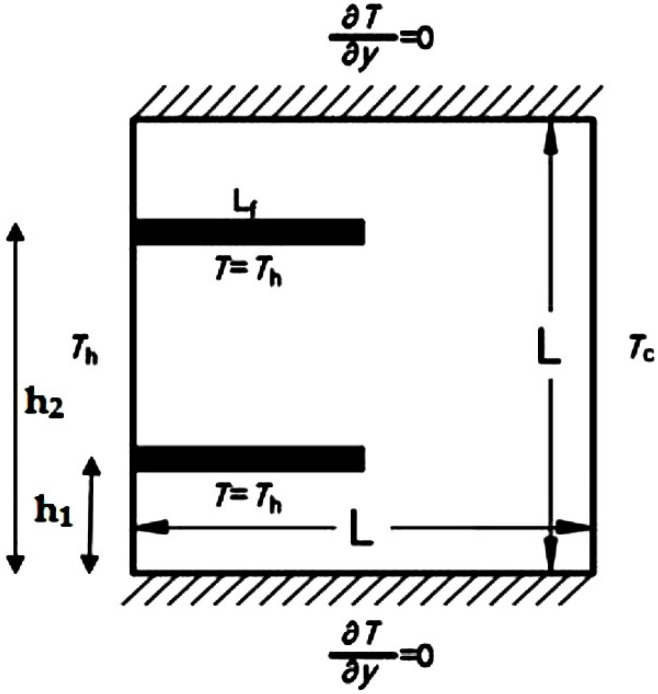
The configuration used for the computational domain.

In this study, a two-dimensional steady state laminar natural convection of air/Al_2_O_3_ nanofluid flow was investigated. Due to a small temperature difference between the hot and cold walls, all of the thermophysical properties of the nanofluid were assumed constant except for the density variation that was modeled using the Boussinesq approximation. Figure 1 represents the geometry of a square cavity of length *L* with two fins at the hot wall. h_1_ represents the position of the lower fin relative to the lower wall while h_2_ represents the upper fin position relative to the lower wall. In the current investigation, both slip and continuum flow regimes were analyzed.

Dispersing nanoparticles to the base fluid will enhance the thermophysical properties of the resulting nanofluid. As reported in Al-Kouz et al. [21], these properties can be calculated based on the following equations:

Viscosity:

(1) $$\mu_{nf}=\frac{\mu_{f}}{{\left(1-\emptyset \right)}^{2.5}}$$

Density:

(2) $$\rho_{nf}=\left(1-\emptyset \right)\rho_{f}+\emptyset \rho_{s}$$

Heat Capacitance:

(3) $$C_{P_{nf}}=\left(1-\emptyset \right){\left(C_{P}\right)}_{f}+\emptyset {\left(C_{P}\right)}_{s}$$

Thermal Expansion Coefficient:

(4) $$\beta_{nf}=\beta_{f}\left[\frac{1}{1+\frac{\left(1-\emptyset \right)\rho_{f}}{\emptyset \rho_{s}}}\frac{\beta_{s}}{\beta_{f}}+\frac{1}{1+\frac{\emptyset }{1-\emptyset }\frac{\rho_{s}}{\rho_{f}}}\right]$$

Thermal Conductivity:

(5) $$k_{nf}=k_{f}\frac{k_{s}+2k_{f}-2\emptyset \left(k_{f}-k_{s}\right)}{k_{s}+2k_{f}+\emptyset \left(k_{f}-k_{s}\right)}$$

Table 1 shows the thermophysical properties utilized to obtain the resulting properties of the Al_2_O_3_-air nanofluid.

The governing equations of the current study are reported in Al-Kouz et al. [21] and are summarized below:

Continuity:

(6) $$\frac{\partial u}{\partial x}+\frac{\partial v}{\partial y}=0$$

*x*-momentum:

(7) $$\rho_{nf}\left(u\frac{\partial u}{\partial x}+v\frac{\partial u}{\partial y}\right)=-\frac{\partial P}{\partial x}+\mu \left(\frac{\partial^{2}u}{\partial x^{2}}+\frac{\partial^{2}u}{\partial y^{2}}\right)$$

*y*-momentum:

(8) $$\rho_{nf}\left(u\frac{\partial v}{\partial x}+v\frac{\partial v}{\partial y}\right)=-\frac{\partial P}{\partial y}-\rho_{nf}g+\mu \left(\frac{\partial^{2}v}{\partial x^{2}}+\frac{\partial^{2}v}{\partial y^{2}}\right)$$

Energy:

(9) $$\rho_{nf}C_{p_{nf}}\left(u\frac{\partial T}{\partial x}+v\frac{\partial T}{\partial y}\right)=k_{nf}\left(\frac{\partial^{2}T}{\partial x^{2}}+\frac{\partial^{2}T}{\partial y^{2}}\right)$$

With the following boundary conditions in the slip flow regime as reported in Karniadakis et al. [43], Lockerby et al. [44] and Colin [45]:

(10) $$u_{w}-u_{g}=\left(\frac{2-\sigma_{v}}{\sigma_{v}}\right)\lambda \frac{\partial u}{\partial n}\approx \left(\frac{2-\sigma_{v}}{\sigma_{v}}\right)K_{n}\left(u_{g}-u_{c}\right)$$

(11) $$v_{g}=0$$

(12) $$T_{w}-T_{g}=\left(\frac{2-\sigma_{T}}{\sigma_{T}}\right)\frac{2\gamma }{\gamma +1}\frac{k}{\mu c_{v}}\lambda \frac{\partial T}{\partial n}\approx \left(\frac{2-\sigma_{T}}{\sigma_{T}}\right)\frac{2\gamma }{\gamma +1}\frac{k}{\mu c_{v}}\left(T_{g}-T_{c}\right)$$

In Equations (10) and (12), $\sigma_{v}$ and $\sigma_{T}$ refer to the momentum and thermal accommodation coefficients, respectively, and *Kn* is defined as:

(13) $$K_{n}=\frac{\lambda }{L}$$

where *L* is the square cavity characteristic length and λ is the mean free path.

The imposed thermal boundary conditions at *x* = 0 and *L*:

(14) $$At\left(x=0,y\right),\;T=T_{h}$$

(15) $$At\left(x=L,y\right),\;T=T_{c}$$

where *T_h_* is the temperature at the hot surface and *T_c_* is the temperature at the cold surface. The temperature of the fins was set to *T_h_*

The local heat fluxes could be calculated by Equations (16) and (17) as reported by [21]:

(16) $$q_{F}^{\prime \prime }=-k\frac{\partial T}{\partial n}{|}_{F}$$

(17) $$q_{h}^{\prime \prime }=-k\frac{\partial T}{\partial n}{|}_{h},q_{c}^{\prime \prime }=-k\frac{\partial T}{\partial n}{|}_{c}$$

To calculate the total heat transfer from the hot to the cold wall, one could integrate the local heat flux along the wall of the hot wall combined with the fins as follows:

(18) $$Q=\sum \left(\int_{A_{h}}q_{h}^{\prime \prime }dA_{h}+\int_{A_{F}}q_{F}^{\prime \prime }dA_{F}\right)=\int_{A_{c}}q_{c}^{\prime \prime }dA_{c}$$

Then, the average heat transfer coefficient along the combined hot wall and the fins or along the cold surface was derived as follows:

(19) $$\bar{h}=\frac{Q}{\left(T_{i}-T_{o}\right)A_{T}}=\frac{Q}{\left(T_{i}-T_{o}\right)A_{c}}$$

From the previous equation, one could derive the average Nusselt number for *L* = 1 m, where:

(20) $$Nu=\frac{\bar{h}L}{k_{n_{f}}}=\frac{\bar{h}}{k_{nf}}$$

Following Parvin et al. [20], the total entropy generation is defined as:

(21) $$Sgen_{tot}=Sgen_{f}+Sgen_{h}$$

In Equation (21), *Sgen_f_* is the entropy generation caused by the flow and *Sgen_h_* is the entropy generation due to heat, where,

(22) $${Sgen}_{f}=\frac{k_{nf}}{{T_{o}}^{2}}\left[{\left(\frac{\partial T}{\partial x}\right)}^{2}+{\left(\frac{\partial T}{\partial y}\right)}^{2}\right]$$

(23) $${Sgen}_{h}=\frac{\mu_{nf}}{T_{o}}\left[2{\left(\frac{\partial u}{\partial x}\right)}^{2}+2{\left(\frac{\partial v}{\partial y}\right)}^{2}+\left(\frac{\partial u}{\partial x}+\frac{\partial v}{\partial y}\right)\right]\;\mathrm{and}\;T_{o}=\frac{T_{h}+T_{c}}{2}$$

### 2.2. Numerical Solution

In this study, a finite volume technique was utilized using ANSYS Fluent software (Version 18, ANSYS, Inc., South pointe, PA, USA) to investigate the flow, heat transfer characteristics and the total entropy generation for steady, 2-D, laminar natural convection rarefied nanofluid in a square cavity. The SIMPLE algorithm presented by Versteeg and Malalasekera [46] and Patankar and Spalding [47] was utilized. In order to calculate the pressure field, the PRESTO algorithm was used. Moreover, a hybrid second order accuracy scheme of upwind and central difference was used to differentiate the convective terms. As a starting point, 40 × 40 mesh elements were tested. In addition, $\sigma_{v}$ and $\sigma_{T}$ for all simulations were considered to be in unity. The solution was converged when the maximum of the normalized absolute residual across all nodes was <10^−6^.

### 2.3. Grid Independency

**Figure 2:**
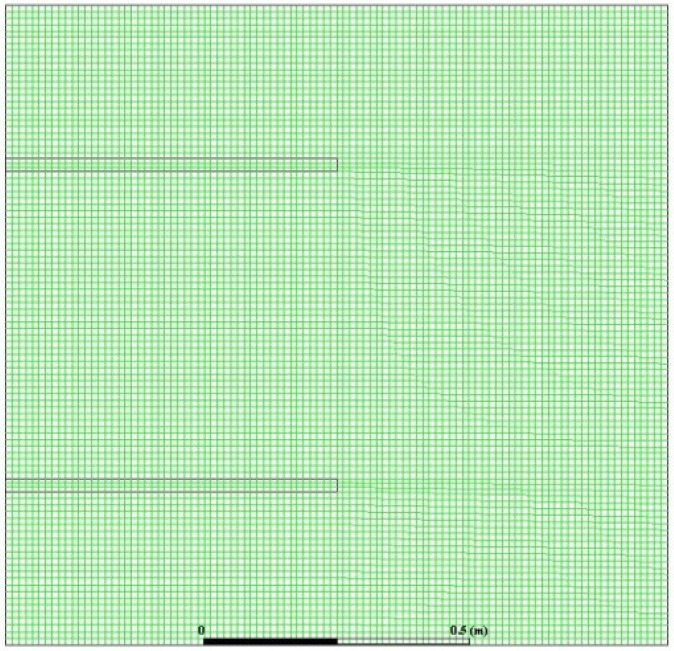
2D mesh utilized in all simulations.

The grid that was used in all simulations was a two dimensional mesh, which is shown in Figure 2. Initially, the step sizes of the grid were increasing in the *x* and *y* directions with expansion factors of 1.06 and 1.15 respectively, these values were selected to capture the gradient’s near solid-fluid interface. Then the mesh was adapted and the velocity gradients near the solid surfaces were calculated. After this, the number of cells was increased to lower the gradients below a certain value. It was noticed that any further change in these parameters would not affect the results. A grid independency test was performed by monitoring *Nu* at the cold surface, and solutions for different numbers of grid nodes were obtained. It was obvious that adding more cells beyond a certain value would not affect *Nu*. In addition, the average magnitude of the velocity inside the cavity was monitored and tabulated. Table 2 summarizes the values of *Nu* as well as the velocity magnitude inside the cavity, along with their relative error to the values obtained for a mesh size of 100 × 100 elements.

**Figure 3:**
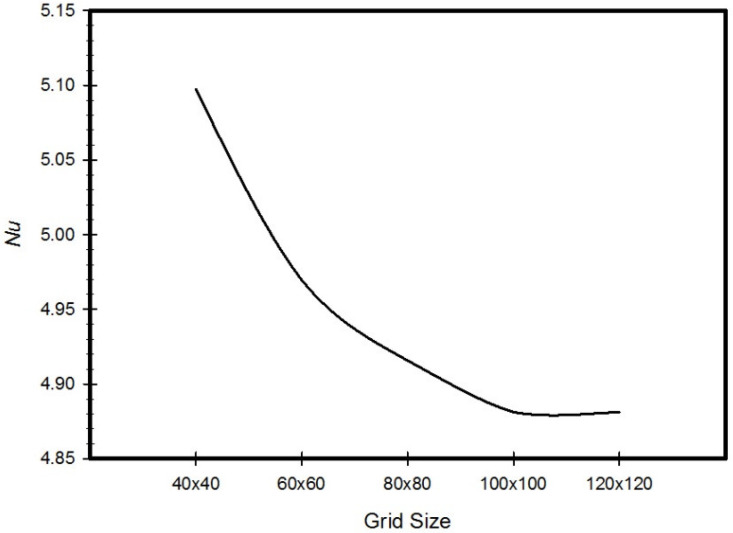
Grid independency test for Nusselt number.

Figure 3 and Table 2 demonstrate that the solution was converged for the 100 × 100 nodes grid size. This grid size was considered for all simulations conducted in this study.

### 2.4. Code Verification

**Figure 4:**
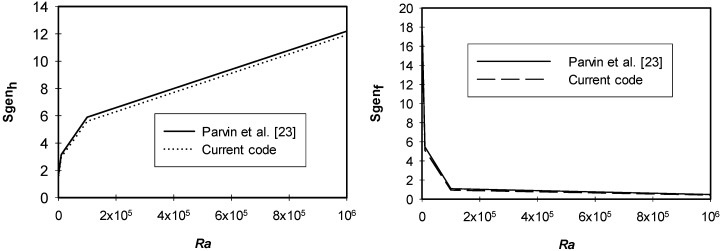
Validation of the current code.

For verification purposes, results of the current code were compared with the results extracted by Parvin et al. [23] for the case of an odd shaped enclosure filled with Cu/water nanofluid. Figure 4 illustrates a satisfying agreement of our proposed model and the model obtained by Parvin et al. [23] at *ϕ* = 5%.

## 3. Results and Discussion

**Figure 5:**
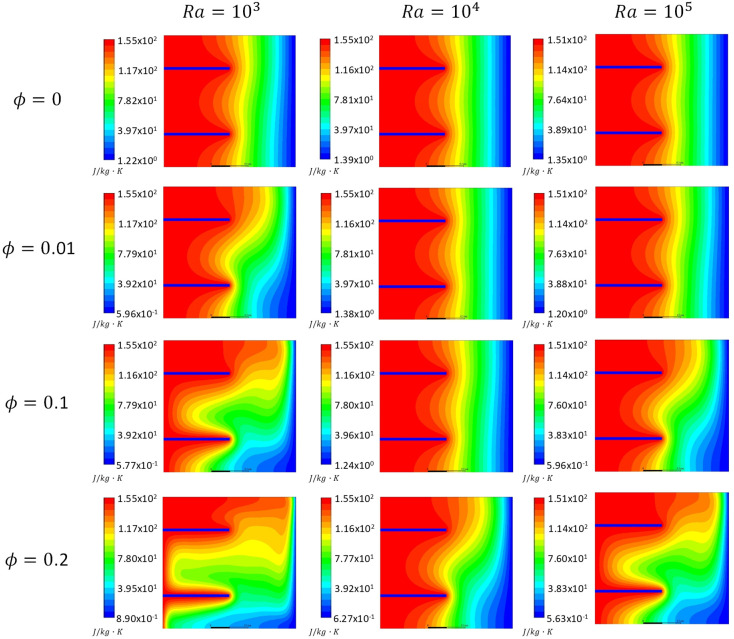
Total entropy generation contours, *Kn* = 0 at different nanoparticles volume fractions (*ϕ* = 0, 0.01, 0.1 and 0.2) and *Ra* = 10^3^, 10^4^ and 10^5^.

**Figure 6:**
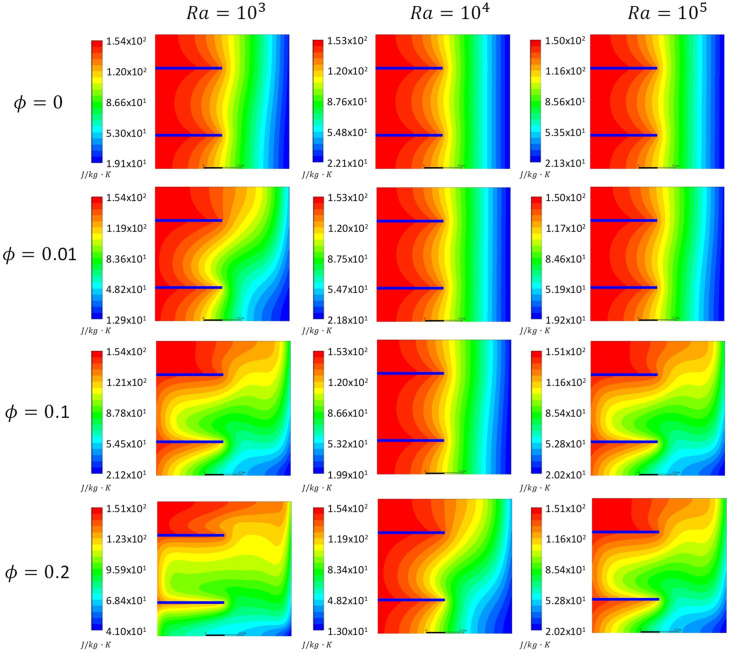
Total entropy generation contours, *Kn* = 0.05 at different nanoparticles volume fractions (*ϕ* = 0, 0.01, 0.1 and 0.2) and *Ra* = 10^3^, 10^4^ and 10^5^.

**Figure 7:**
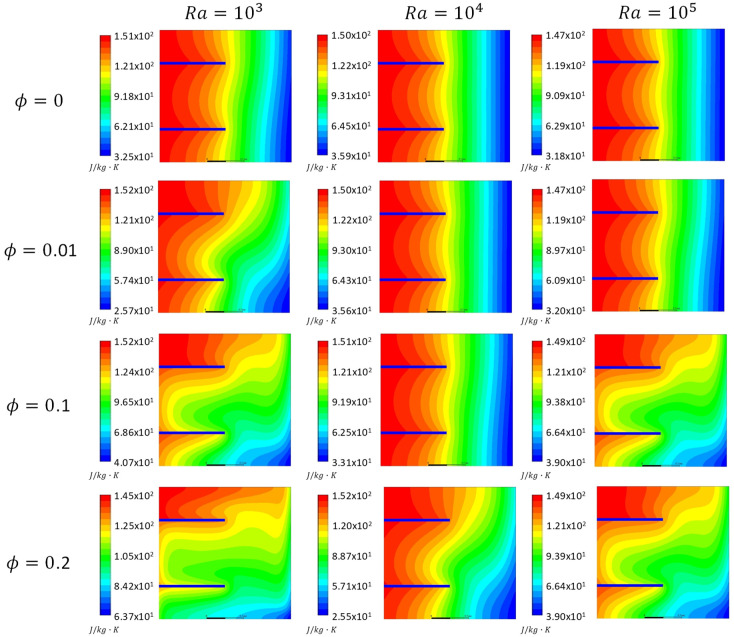
Total entropy generation contours, *Kn* = 0.1 at different nanoparticles volume fractions (*ϕ* = 0, 0.01, 0.1 and 0.2) and *Ra* = 10^3^, 10^4^ and 10^5^.

Figure 5, Figure 6 and Figure 7 show the total entropy generation contours inside the square cavities with a fin position of *H_F_* = 0.25, 0.75 m and fin lengths of 0.5 m for the cases where *Kn* = 0, 0.05 and 0.1 to cover both the slip and continuum flow regimes. Moreover, *ϕ* = 0, 0.01, 0.1 and 0.2 were considered. The contours were plotted for cases where *Ra* = 10^3^, 10^4^ and 10^5^. It was clear from the contours that there was a formation of a large clockwise rotating cell. By increasing *Kn* for the same *ϕ* and *Ra*, less circulation is observed inside the cavity. This decrease will affect the heat transfer characteristics. For the cases of *ϕ* = 0.2 and different *Kn*, more distortion to the flow was observed compared to the other values of ϕ. More recirculation and distorted contours lead to better heat transfer enhancement. Moreover, figures showed that as *Ra* increased for the same *Kn* and *ϕ*, more distortion of the contours occurred inside the cavity, and hence entropy generation increased as a consequence to the increase in the velocity gradients. But still the entropy generation caused by heat was dominant.

**Figure 8:**
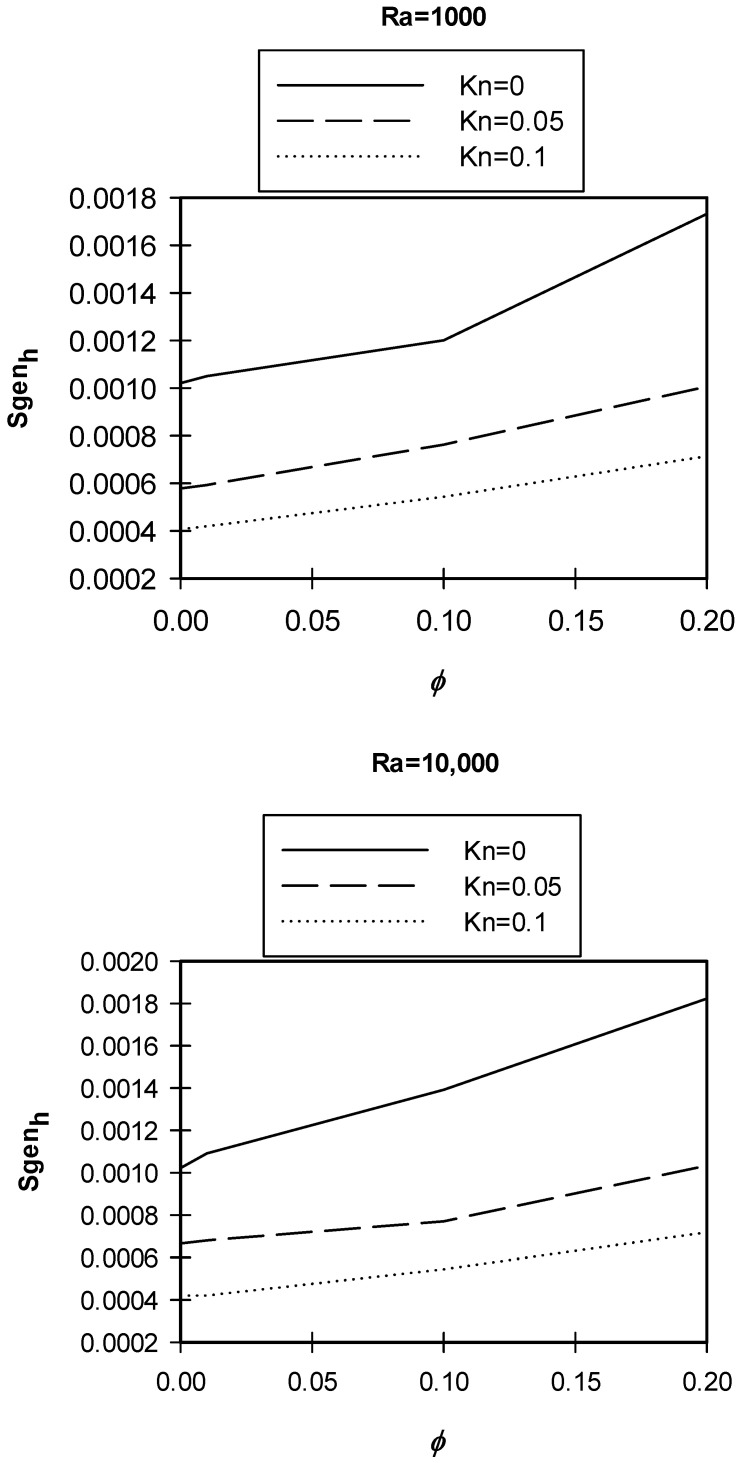
Variation of *Sgen_h_* with φ at different *Kn*, *Ra* = 1000, 10,000.

Figure 8 illustrates variations in entropy generation caused by heat for different nano solid particles volume fractions at different *Kn* values for the cases of *Ra* = 1000 and 10,000. The graphs show that for the two values of *Ra*, as the ϕ increased the entropy generation due to heat increased as well. This could be attributed to the fact that at a low *Ra*, the dominant mode of heat transfer was conduction, and by adding nano solid particles, *k*_eff_ would increase and a better heat transfer was achieved. Better heat transfer implied that the increase in the entropy generation resulted from heat. Moreover, the graphs showed that as *Kn* increased for the same ϕ, the entropy generation due to heat decreased. Higher *Kn* resulted in more rarefaction effects and consequently less interaction between the nanofluid particles, which lead to less entropy generation. Finally, the graphs also showed that for the higher *Ra*, the entropy generation due to heat increased for the same values of *Kn* and the nano solid particles volume fraction. In Figure 5, Figure 6, Figure 7 and Figure 8, as *Ra* increased, convection became the dominant mode of heat transfer leading to greater circulation of the flow, and consequently an increase in the total entropy generation was observed.

**Figure 9:**
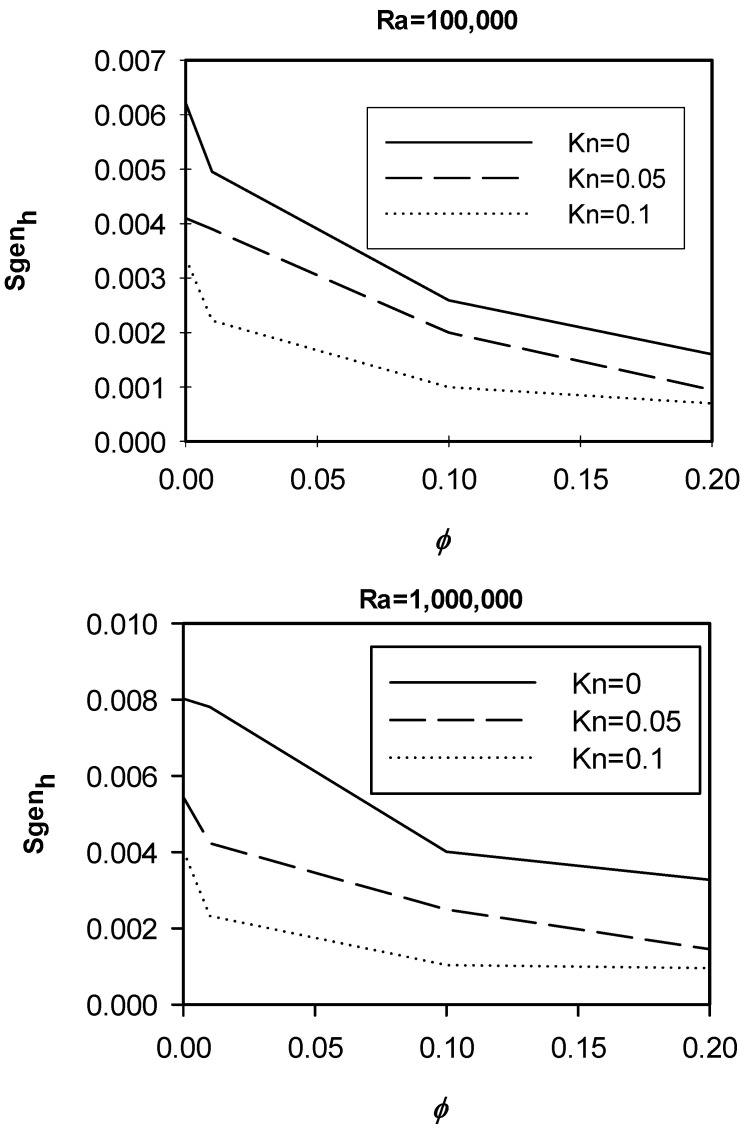
Variation of *Sgen_h_* with ϕ at different *Kn*, *Ra* = 100,000, 1,000,000.

Figure 9 shows variations of the entropy generation due to heat for different nano solid particles volume fractions at different *Kn* for the cases of *Ra* = 10^5^ and 10^6^. The graphs show that for the two values of *Ra*, as the ϕ increased the entropy generation caused by heat decreased. This was mainly because at high *Ra*, the dominant mode of heat transfer was convection, and by adding nano solid particles, the lowering effect of nano solid particles on convection heat transfer became dominant. Moreover, the graphs show that a higher *Kn* would result in less entropy generation. Finally, the graphs also show that for the higher *Ra*, the entropy generation as a result of heat increased for the same value of *Kn* and *ϕ*.

**Figure 10:**
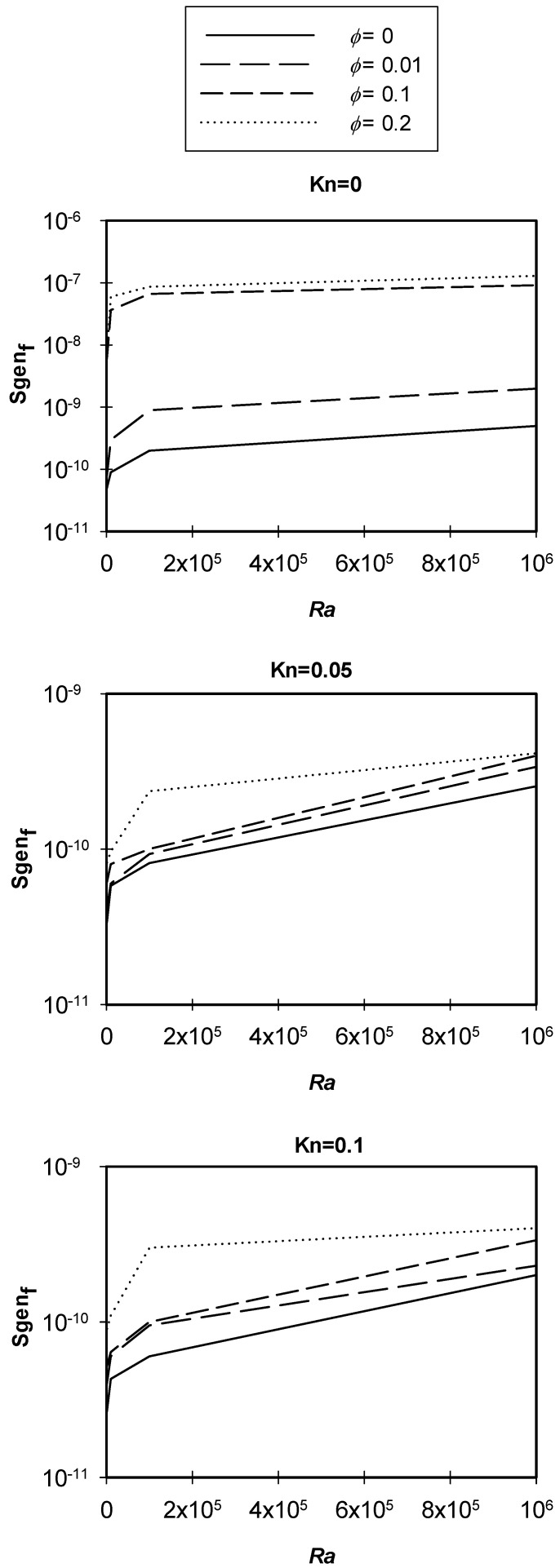
Variation of *Sgen_f_* with *Ra* at different φ, *Kn* = 0, 0.05 and 0.1.

Variations of the entropy generation attributable to the flow with *Ra* at different values of nano solid particles volume fractions are plotted in Figure 10. The graph shows that as *Ra* increased, the entropy generation by reason of the flow would increase. As *Ra* increased, more circulation occurred inside the cavity, which resulted in an increase in both velocity gradient and entropy generation. Moreover, as *Kn* increased, the entropy generation due to flow would decrease, as a result of the rarefaction effects. Finally, as the nano solid particles volume fraction increased, the entropy generation increased because of the flow (friction) effects.

Based on the simulation results, a correlation of the entropy generation among all parameters considered in this study with R^2^ = 0.92 was presented as follows:

(24) $$S_{gen,tot}=C_{1}C_{2}^{Kn}Ra^{C_{3}}C_{4}^{\phi }$$

where, *C*_1_ = 2.2 × 10^−4^ kJ/kg∙K, *C*_2_ = 0.134, *C*_3_ = 0.226, *C*_4_ = 0.0077.

It is obvious that the Bejan number was close to unity for all simulations conducted in the study. The Bejan number (*Be*) is defined as follows:

(25) $$Be=\frac{{Sgen}_{h}}{{Sgen}_{tot}}$$

**Figure 11:**
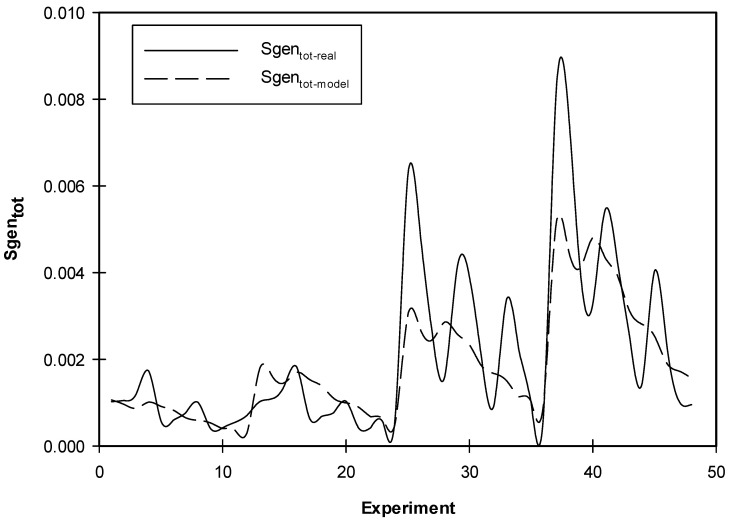
Comparison between the *Sgen_tot_* obtained by simulations and those obtained by correlations.

Figure 11 shows a comparison between the total entropy generation results obtained from the simulations with those obtained from the correlations, the figure shows that there was a great match between the simulation and the correlation results. Deviations between the two were noticed for the conditions at which *Kn* = 0 and *ϕ* = 0.

**Figure 12:**
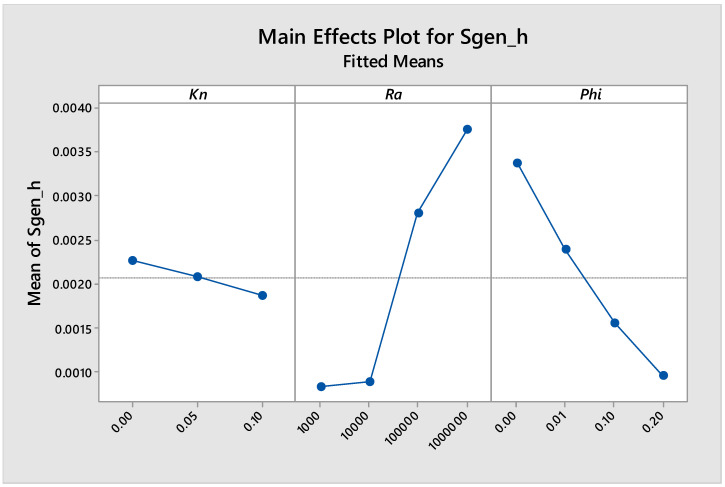
Main effects plot for *Sgen_h_* vs. *Kn*, *Ra* and *ϕ*.

**Figure 13:**
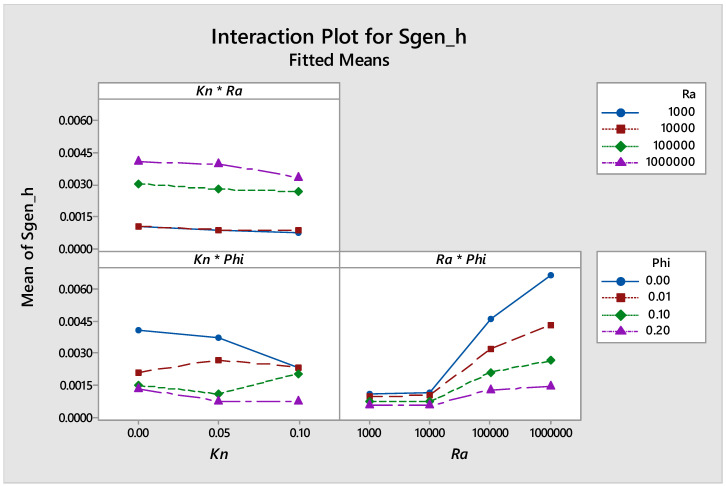
Interaction plot for *Sgen_h_*.

**Figure 14:**
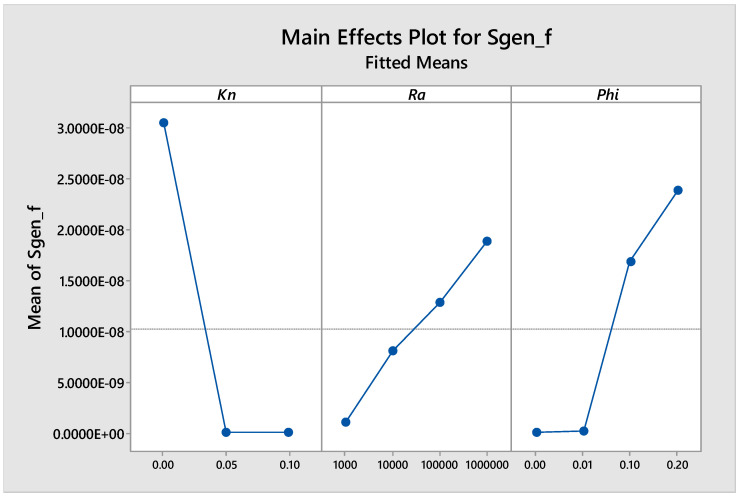
Main effects plot for *Sgen_f_* vs. *Kn*, *Ra* and *ϕ*.

**Figure 15:**
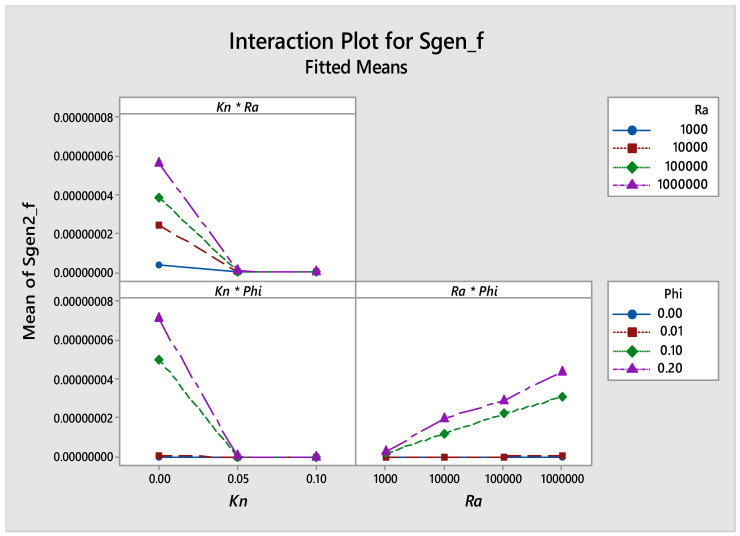
Interaction plot for *Sgen_f_*.

Using Minitab software (Version 18, minitab, State college, PA, USA), the design of experiments for the simulations conducted in this work is shown in Figure 12, Figure 13, Figure 14 and Figure 15. Figure 12 shows the main effects of *Kn*, *Ra* and *ϕ* on the entropy generation due to heat. It was clear from the figure that there was a strong direct proportional relationship between *Sgen_h_* and *Ra* higher than 10^4^, and a weak proportional relationship for *Ra* less than 10^4^. The graph also shows that there was a weak inverse proportional relationship between *Sgen_h_* and *Kn*. Moreover, the graph shows a strong inverse proportional relationship between *Sgen_h_* and *ϕ*.

Figure 13 shows the interaction plots between parameters investigated in this work on *Sgen_h_*, and the graph shows that there was an interaction between *Ra* and *ϕ*, as they intersect. The changes that we were getting at the level of one independent variable was not changing systematically across the levels of the other independent variable. Therefore, a special effect was achieved when combining them, which was in harmony with the opposite trends seen in Figure 8 and Figure 9.

Figure 14 shows the main effects of (*Kn*, *Ra* and *ϕ*) on the entropy generation due to flow, and it is clear from the figure that there was a strong direct proportional relationship between *Sgen_f_* and *Ra*. The graph also shows that there was a strong inverse proportional relationship between *Sgen_f_* and *Kn* values less than 0.05. For *Kn* values greater than 0.05, there was no effect on *Sgen_f_*. Moreover, the graph shows a strong direct proportional relationship between *Sgen_f_* and the volume fraction of the nano solid particles for volume fractions greater than 0.01, and there was almost negligible effect for volume fractions less than 0.01.

Figure 15 illustrates the interaction plots between parameters investigated in this work on *Sgen_f_*, the graph shows that there was an interaction between any two parameters, as they do intersect.

Finally, in order to find the conditions at which the minimum entropy generation was obtained, an optimization of the multi variable function of the total entropy generation for the parameter ranges considered in the study was conducted. The optimization that yielded the minimum total entropy generation revealed that this would happen at *Ra* = 1001.1, *ϕ* = 0.19995 and *Kn* = 0.099 with minimum total entropy generation of 3.29 × 10^−4^ kJ/kg∙K.

It is worth mentioning here that another simulation with the resulting conditions for the minimal entropy generation obtained from the optimization was carried out and the total entropy generation was calculated and was equal to 3.2645 × 10^−4^ kJ/kg∙K, which makes the optimal value extracted from the optimization of the model’s correlation in great agreement with the experimental simulation value.

## 4. Conclusions

Entropy generation analysis using CFD for a steady state, two-dimensional low-pressure gaseous laminar nanofluid flow inside a square cavity equipped with two solid fins attached to the hot wall was carried out. Such flows are of great importance due to their engineering applications. Rarefaction, *Ra* and *ϕ* effects on entropy generation were investigated. Results showed that:

1. As *Kn* increased, entropy generation decreased.
2. For low *Ra* numbers, the entropy generation due to flow increased as *ϕ* increased.
3. For higher *Ra*, the entropy generation due to flow decreased as *ϕ* increased.
4. The entropy generation due to heat increased as both *Ra* and *ϕ* increase.
5. A correlation model of the total entropy generation as a function of all the parameters investigated in this study was proposed.
6. The conditions for the optimum (minimum) entropy generation in the investigated ranges of the parameters in this study were calculated mathematically and were validated numerically using CFD.

## Figures and Tables

**Table 1:** Thermophysical properties of air and Al_2_O_3_.

Physical Properties	Air	Al_2_O_3_
*Cp* (J/kg∙K)	1006.43	765
*ρ* (kg/m^3^)	1	3970
*k* (W/m^2∙^K)	0.025	40
*β* (1/K)	0.00333	0.0000085
*α* (m^2^/s)	0.000019	0.00001317

**Table 2:** Mesh independency test.

Mesh Size	Average Velocity (m/s)	Relative Error in the Average Velocity (%)	*Nu*	Relative Error in *Nu* (%)	Simulation Time (s)
40 × 40	0.00055464421	0.050516	5.0973	0.044250507	415
60 × 60	0.00057143194	0.021778	4.9694	0.01804847	622
80 × 80	0.00057617336	0.013661	4.9157	0.00704730	739
100 × 100	0.00058415352	0.0	4.8813	0.0	823
120 × 120	0.00058415352	0.0	4.881297	6.15 × 10^−7^	876

## Nomenclature

The following notations are used in this manuscript:

**Notations**	
*A_c_*	Cold wall area (m^2^)
*A_h_*	Hot wall area (m^2^)
*A_F_*	Fin area (m^2^)
*Be*	Bejan number
*C_p_*	Specific heat (J∙kg^−1^∙K^−1^)
*G*	Gravity acceleration in the x direction (m/s^2^)
*h*_1_	Fin 1 Position (m)
*h*_2_	Fin 2 Position (m)
*h*	Convection heat transfer coefficient
*K*	Thermal conductivity (W∙m^−1^∙K^−1^)
*Kn*	Knudsen number
$k_{f}$	Fluid thermal conductivity (W∙m^−1^∙K^−1^)
$k_{nf}$	Nanofluid thermal conductivity (W∙m^−1^∙K^−1^)
$k_{s}$	Nano particles thermal conductivity (W∙m^−1^∙K^−1^)
*L*	Length of the square cavity (m)
L_F_	Fin length (m)
*Nu*	Nusselt Number
*P*	Pressure (Pa)
*Q*	Heat transfer (W)
$q_{c}^{\prime \prime }$	Local heat flux at the wall of the cold surface (W/m^2^)
$q_{h}^{\prime \prime }$	Local heat flux at the wall of the hot surface (W/m^2^)
$q_{F}^{\prime \prime }$	Local heat flux at the fin (W/m^2^)
*R*	Universal gas constant (J/mol∙K)
*Ra*	Rayleigh number (*gβ*(*T*_1_-*T*_2_)*L*3/*αν*)
*T*	Temperature (°C)
*T_c_*	Temperature of the first cell from the wall (°C)
*T_i_*	Hot surface temperature (°C)
*T_o_*	Cold surface temperature (°C)
*T_g_*	Temperature of the nanofluid (°C)
*T_w_*	Temperature of the wall (°C)
*u*	Velocity in x-direction (m/s)
*u_c_*	Tangential velocity of the first cell from the wall (m/s)
*u_g_*	Tangential velocity of the nanofluid (m/s)
*u_w_*	Tangential velocity of the wall (m/s)
*V*	Velocity in y-direction (m/s)
*x*, *y*	Cartesian coordinates [m)
**Greek Symbols**	
*α*	Thermal diffusivity (m^2^/s)
*β*	Thermal expansion coefficient (1/K)
*γ*	Specific weight (N/m^3^)
*λ*	Molecular mean free path (m)
*μ*	Dynamic viscosity (kg∙m^−1^∙s^−1^)
*ν*	Kinematic viscosity (m^2^∙s^−1^)
*ϕ*	Nano particles volume fraction (%)
*ρ*	Density of air, given by ideal gas equation (P/RT), (Kg/m^3^)
*σ_T_*	Thermal accommodation coefficient
*σ_v_*	Momentum accommodation coefficient
**Subscripts**	
*Eff*	Effective
*f*	Fluid
*F*	Fin
*g*	Gas flow
*i*	Hot wall
*n*	Normal
*nf*	Nanofluid
*o*	Cold wall
*r*	Ratio
*w*	Wall
